# Some Facts about the Automatic Mallet

**Published:** 1897-01

**Authors:** W. G. Laine

**Affiliations:** Munich, Germany.


ARTICLE II.

### SOME FACTS ABOUT THE AUTOMATIC MALLET.

DR. W. G. LAINE, MUNICH, GERMANY.

There is no mallet-system but has its adherents, assuring us that the system employed by them possesses the greatest advantages. In view of this fact I desire to state, that it is merely a feeling of gratitude which after a nine years' practice prompts me to say something in favor of the Automatic Mallet. Excepting the engine there is not an instrument in our professional line that has been of such signal service to me as the mallet named. I have given most of the other systems a trial but have always fared best with the Automatic Mallet. Although firmly persuaded that the best of fillings may be executed with any mallet, the Automatic Mallet has always been the instrument with which I have recorded the greatest number of successful fillings.

Having been compelled last winter to interrupt my practice in consequence of a protracted illness, I took occasion during an eight months' journey, to speak with some of my professional friends about the automatic mallet and its advantages.

There are a number of dentists in Europe as well as in America, who willingly admit the advisability of the Automatic Mallet for the treatment of ordinary cavities but hold that it is not adapted for large and difficult contour fillings. Recently one of our dental journals contained the assertion, that the Automatic Mallet was not to be met with in the operating rooms of "really fine gold-fillers." This statement will be disputed or conceded accordingly as the dentist travelling about chooses to study the doings of his professional friends attentively or in a general and more or less superficial way. The Automatic Mallet is indeed not the proper instrument for a physically weak operator since the exertion or more correctly stated the expenditure of force which it entails is not to be deemed inconsiderable. Besides it requires a greater amount of attention on the part of the operator than any other system. If this attention is lacking, the tooth is very easily fractured and a part broken off. This is a disadvantage, I frankly admit. But what do the advantages consist in? If I employ the Automatic Mallet I do not require a battery, an excavating machine or an assistant. This to be sure is worth a great deal. The stroke comes down exactly in the moment and exactly with the amount of force I desire, and the power of condensation is exceedingly great. The plugger and mallet being combined in one and the same instrument I am never at a loss as to a quick procedure in the case of difficult cavities nor is there any danger of the conducting posts, the cable of the engine, etc., knocking down the alcohol-lamp or other objects from the bracket.

These in a general way are the advantages of the automatic system. The question next suggests itself whether an operator employing this mallet works more slowly than an operator using a different system. There is no doubt but what a dentist employing the automatic mallet will never attain the speed Dr. Bonwill records with his machine-mallet. But let us put the question frankly: How many dentists are there that can stand a comparison with Dr. Bonwill in the line of speedy work? Very few indeed, I think. This does not go to say, however, that an operator may not do quick filling-work in using the automatic mallet. The rate will more or less depend on the personal skill of the operator. In the course of practice we are taught to avail ourselves of a number of expedients. Thus in supporting the plugger with a finger of the left hand we may easily score 300 strokes in a minute. Supposing a stroke of the automatic mallet to be equal to one pound only, 300 strokes equal a pressure of 300 lbs., quite assuredly a very considerable power.

I have often asked dentists who asserted that difficult contour fillings could not be made with the automatic mallet, to assign some reason for their assumption but have either been told in a general way that the fault lay in the system, or received an answer that completely evaded the point.

The statement, that eminent dentists do not employ the automatic mallet is certainly erroneous. To disprove it I need only mention the names of some prominent dentists just occurring to me in this moment as Dr. Northrup, Dr. Barett, Dr. Abbott, Dr. Flesh, Dr. Sandre and Dr. Thomas.

I might also be permitted to add the name of a friend of mine who unfortunately is no longer among the living of our profession, viz: Dr. Alexowicz of Vienna, court-dentist of the late Emperor of Brazil. Dr. Alexowicz was of a sickly constitution and moreover not an adherent of contour-fillings. Owing to his illness he worked very little with gold. Still I have never, neither during the six years whilst I worked as assistant in the operating rooms of prominent dentists nor in the course of my own practice met with more difficult gold fillings than those which Dr. Alexowicz showed me.

Another professional friend of mine, whose reputation as a gold-filler, however, in contradistinction to the former, is well known, ought to be referred to here, viz: Dr. Sandre. The latter showed me some fillings he made in his practice and which I regard to be the largest fillings that can be conceived of as possible. All were made with the automatic mallet.

If names such as these can be mentioned in favor of cur mallet, the statement, that the latter is not used by our prominent dentists, requires no further refutation. I would, however, beg leave to add once more, that the best and most beautiful contour fillings may be executed with any of our mallet systems or as Dr. Ottolengui in his "Methods of Filling Teeth" very appropriately says: "In practice a broken instrument is as good as any in the hand of the skillful." It is and ever will be the man behind the instrument, who does the work. This is a principle of our profession, that will always hold good.

The aim of these lines has been to prevent superficial criticism from prejudicing the views of beginners. The automatic mallet is my favorite instrument and I shall ever be ready to give it the legitimate amount of commendation, it deserves.

Lastly as regards the question: which mallet-system is most in use? I venture to say that if the dealers' books of sale were consulted the answer would be: the Automatic Mallet.

